# Towards a High-Power Si@graphite Anode for Lithium Ion Batteries through a Wet Ball Milling Process

**DOI:** 10.3390/molecules25112494

**Published:** 2020-05-27

**Authors:** Marta Cabello, Emanuele Gucciardi, Alvaro Herrán, Daniel Carriazo, Aitor Villaverde, Teófilo Rojo

**Affiliations:** 1 Centre for Cooperative Research on Alternative Energies (CIC energiGUNE), Basque Research and Technology Alliance (BRTA) Parque Tecnológico de Álava, 01510 Miñano, Álava, Spain; egucciardi@cicenergigune.com (E.G.); aherran@cicenergigune.com (A.H.); dcarriazo@cicenergigune.com (D.C.); avillaverde@cicenergigune.com (A.V.); teo.rojo@ehu.eus (T.R.); 2 IKERBASQUE, Basque Foundation for Science, 48013 Bilbao, Spain; 3 Inorganic Chemistry Department, Faculty of Science and Technology, University of the Basque Country UPV/EHU, 48080 Bilbao, Spain

**Keywords:** silicon, graphite, ball milling, alloying anodes, lithium ion batteries

## Abstract

Silicon-based anodes are extensively studied as an alternative to graphite for lithium ion batteries. However, silicon particles suffer larges changes in their volume (about 280%) during cycling, which lead to particles cracking and breakage of the solid electrolyte interphase. This process induces continuous irreversible electrolyte decomposition that strongly reduces the battery life. In this research work, different silicon@graphite anodes have been prepared through a facile and scalable ball milling synthesis and have been tested in lithium batteries. The morphology and structure of the different samples have been studied using X-ray diffraction, X-ray photoelectron spectroscopy, Raman spectroscopy, and scanning and transmission electron microscopy. We show how the incorporation of an organic solvent in the synthesis procedure prevents particles agglomeration and leads to a suitable distribution of particles and intimate contact between them. Moreover, the importance of the microstructure of the obtained silicon@graphite electrodes is pointed out. The silicon@graphite anode resulted from the wet ball milling route, which presents capacity values of 850 mA h/g and excellent capacity retention at high current density (≈800 mA h/g at 5 A/g).

## 1. Introduction

The request for high energy storage systems capable of reaching the customer expectations in various markets fields (EVs, mobile phones industries, computers) is increasing year after year and, among other battery technologies, lithium-ion batteries (LIBs) are the most attractive topic in energy storage research in recent years. Currently, Li-ion technology is continuously developing, trying to fill the gap between batteries and the new technology systems, which are increasingly seeking more power and more energy. Technology’s development rate is much faster than that of batteries. For this reason, remarkable research studies have been focused on the study of new systems (solid state batteries, Li-sulphur, Polymers Li-ion batteries) [1,2,3] and new materials for LIBs [4] with the aim to improve not only safety and energy density but also the cycle life of LIBs.

Commonly used anodes for LIBs are based on graphite that shows a gravimetric capacity of about 372 mA h/g [5] Additionally, hard carbon, soft carbon, microbeads carbon, lithium titanate, and, likewise, the previously mentioned graphite, are classified as insertion type materials. Another group of negative electrodes showing very high capacity values is the Li-alloy anodes. The alloying and de-alloying processes take place by a multiple electron exchange mechanism that can explain the high capacity reached from these materials [6]. In this context, silicon is one of the most promising negative electrodes for a new generation of LIBs. A lot of interest has been addressed to the Si anode for LIBs for a long time [7,8,9,10,11]. Si is considered an important alternative to graphitic carbon as a negative electrode in LIBs and shows a theoretical capacity of 3579 mA h/g [10]. However, silicon particles suffer larges changes in their volume [10] during battery cycling. Furthermore, the huge volume expansion results in particles cracking and pulverization that leads to the breakage of the solid electrolyte interphase (SEI), which induces continuous irreversible electrolyte decomposition. This strongly reduces battery life and make its use difficult in real systems. Furthermore, continuous SEI formation causes a large Li consumption that, in real systems, require an oversized cathode [12,13,14]. This constant Li loss in the anode side drives to a battery with a poor coulombic efficiency since the accumulation of SEI clogs Li-ion transport. In addition, silicon is a material with poor electronic conductivity.

In order to improve the electrochemical characteristics and performance of Si-based anodes, various strategies have been developed over the years. For instance, with the purpose to overcome the problems due to volume expansion, different approaches have been designed, by means of materials’ nanostructuration [4,6,15,16], deposition of protective layers [17,18], or synthesis of nanocomposites [19,20]. Others research groups, with the aim not only to improve electronic conductivity of Si-based anodes but also to buffer the continuous volume changes and to avoid direct contact with the electrolyte, have proposed to use graphene [21,22,23,24], nanotubes, or graphite shells [4,25,26,27] to encapsulate silicon.

Concerning graphene, despite its application as a pure electrode for LIBs, is still controversial [28]. It was demonstrated that, when used in a composite electrode, it can be beneficial for the improvement of electronical and morphological characteristics. In fact, it was demonstrated that a possible formation of a conductive matrix can possibly buffer the volume changes during charge and discharge or improve the capacity retention as well [29,30].

It is also crucial to emphasize the relevance that the studies on electrolyte additives have contributed to improving battery performances. It was demonstrated that the use of carbonate-based additives helps to achieve a better quality SEI with an improved coulombic efficiency and a long cycling life [31,32,33,34,35,36]. Furthermore, plenty of research has been addressed on binder materials in which the function is to give a stronger adhesion to avoid electrical disconnection between the particles and the current collector. Water-based binders such as poly (acrylic acid) (PAA), alginate, and carboxymethyl cellulose (CMC) and polymers that can form a crosslinked network like styrene butadiene rubber (SBR) are widely investigated, since they are able to accommodate the electrode volumes changes during charge and discharge [32,37,38,39,40,41].

Considering the state-of-the-art improvement of Si-based anodes in this research work, we have followed a very common, simple, and low cost method for obtaining a silicon@graphite (Si@G) sample, which, in combination with few layer graphene (FLG), lithium poly-acrylic acid (Li-PAA), and using an electrolyte with alkylcarbonates additives like fluoroethylene carbonate (FEC) and vinylene carbonate (VC), is able to cycle at high current densities due to the optimal electrode’s structure and morphology.

## 2. Results and Discussion

### 2.1. Composition and Morphology

**Figure 1:**
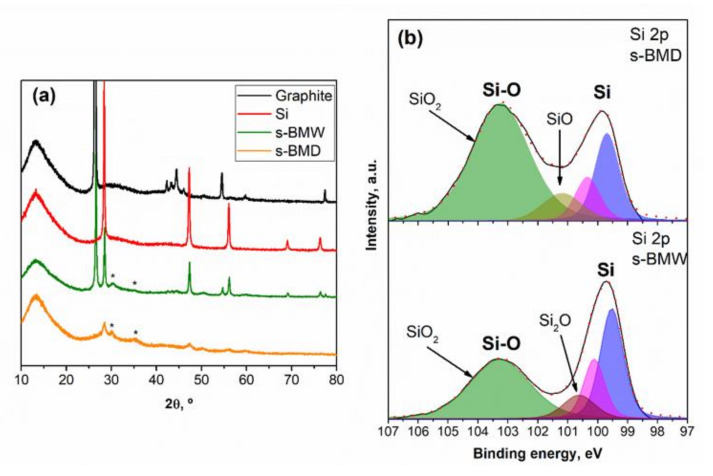
(**a**) X-ray diffraction (XRD) patterns of silicon (Alfa Aesar), graphite, and ball milled samples (s-BMW and s-BMD). The asterisk represents SiO_2_. (**b**) X-ray photoelectron spectroscopy (XPS) spectra of the Si 2p region dry milled sample (s-BMD) and wet milled sample (s-BMW). Colors: blue and magenta (Si2p_3/2_ and Si2p_1/2_, respectively), green (SiO_2_), dark yellow (SiO), and purple (Si_2_O).

The X-ray diffraction (XRD) patterns registered for Si, graphite, and the as-prepared Si@G powders obtained through dry ball milling (hereafter denoted as s-BMD) and wet ball milling (s-BMW) are shown in Figure 1a. All of them show a broad band between 10–20° that corresponds to the sample holder. s-BMW exhibits well-defined diffraction peaks ascribed to the presence of Si (COD 9011998) and graphite (COD 9012230). In addition, two diffraction peaks at 30.4° and 35.5° are assigned to SiO_2_ (marked with an asterisk, COD 4124071) and can be observed in both samples. This fact can be attributed to the presence of air inside the milling bowl that may result in the formation of oxides, among other compounds. s-BMD has a poorly crystalline structure and the Si diffraction peak at 28° is much broader due to the crystallite size decreases while there is an increase of the lattice strain (Table S1). This is in agreement with the results of Gauthier et al. [42]. The graphite diffraction peaks do not appear. Tiwari et al. [43] suggest that the absence of graphite peaks may be attributed to the following reasons: (i) the carbon atoms occupy interstitial positions, (ii) thin graphite layer stick into inter-grain boundaries of major grains, or (iii) amorphization of graphite layers. In order to understand the absence of the graphite peaks, graphite was dry milled (hereafter denoted as M-G) following the same procedure than for s-BMD. The XRD pattern of M-G (Figure S1) shows that graphite is amorphized under these milling conditions. This result is in agreement with Boldyrev and Tkáčová [44] who reported that the high energy ball milling can lead to lattice deformation and material amorphization.

The presence of SiO_2_ in the as-prepared samples was also confirmed by X-ray photoelectron spectroscopy (XPS). Figure 1b displays the XPS spectra of Si 2p region. In order to deconvolute the peaks, Si2p_3/2_ and Si2p_1/2_ have been taken into account, which correspond to Si-Si bonds with a splitting of 0.6 eV. In the spectra, we can differentiate bulk silicon with signals at 99.5 eV (Si 2p_3/2_) and at 100.1 eV (Si 2p_1/2_) and the signal at 103.3 eV arisen from Si−O bonds of SiO_2_. In addition, a signal at 101.3 eV attributed to SiO can be appreciated in the case of s-BMD and a signal at 100.6 eV corresponding to Si_2_O is present in s-BMW. Thus, the SiO_x_ content in s-BMD is 70.49% while, in s-BMW, is 53.28%. Nevertheless, it has to be considered that this technique only provides information of the surface of the material studied.

**Figure 2:**
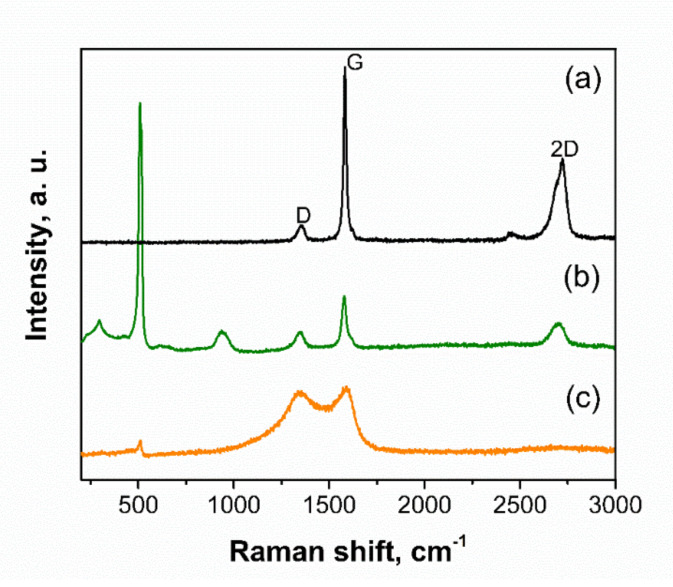
Raman spectra of (**a**) graphite, (**b**) s-BMW, and (**c**) s-BMD.

The Raman spectrum of pristine graphite (Figure 2a) shows the characteristic G band at 1582 cm^−1^, which corresponds to ordered sp^2^ bonded carbon. At 1350 cm^−1^, the D band appears, which is related to defects in the structure. The ratio of the intensities of the D and G bands (R = ID/IG) gives a value close to 0.1, which points out the high graphitic character of this sample. The 2D band at 2720 cm^−1^ is formed by two components and it is asymmetric. As can be seen, the Raman spectrum of s-BMW (Figure 2b) shows the one-phonon peak at 515 cm^−1^ attributed to crystalline Si. Moreover, two-phonon peaks rise at 304 and 970 cm^−1^ and are assigned to silicon overtones [45]. In addition, the D band is enhanced, which suggests an increased disorder likely due to the exfoliation of graphite to platelets that takes place during the milling process. In this case, the 2D band becomes more symmetric, which is characteristic of graphene, even though, considering the value of the full width at half maximum (FWHM) of the 2D band (79 cm^−1^), it can be estimated that the number of graphene layers are more than five [46]. On the contrary, in s-BMD spectrum (Figure 2c), the intensity of the Si peaks decreases and the 2D band cannot be appreciated. Notably, R = 0.95 signifies a high degree of amorphization, which is in agreement with the XRD results previously described. Additionally, the Raman spectrum of M-G was registered (Figure S2) confirming that the dry milling conditions used in this work lead to an amorphization of graphite.

**Figure 3:**
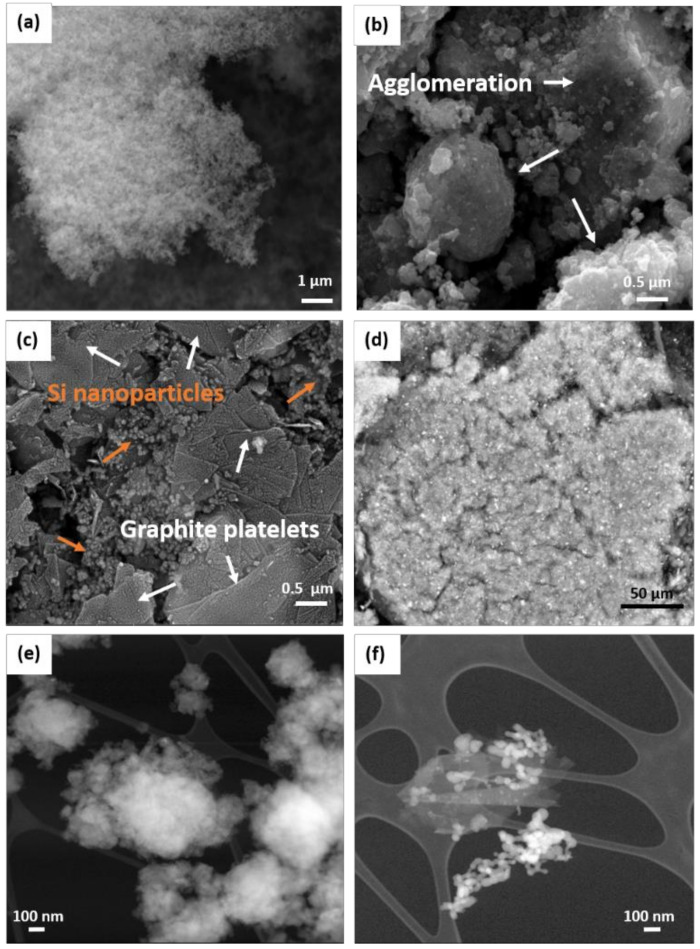
Scanning electron microscopy (SEM) micrographs of (**a**) Si nanoparticles (Alfa Aesar), (**b**) s-BMD, (**c**) s-BMW, (**d**) s-BMW backscattered image. Transmission electron microscopy (TEM) micrographs in dark field of (**e**) s-BMD and (**f**) s-BMW.

The silicon powder from Alfa Aesar is formed by very small particles between 10 and 50 nm as can be seen in the scanning electron microscopy (SEM) image (Figure 3a). The SEM image registered for the s-BMD sample (Figure 3b) suggests that graphite particles were pulverized during the milling process and the silicon nanoparticles are found deposited on the surface of the powdered graphite. Si nanoparticles have a great tendency to agglomerate, which is acknowledged in the same figure. SEM images of graphite before and after milling (M-G) are shown in Figure S3, revealing the pulverization of graphite particles. Figure 3c displays the drastic change in s-BMW where the addition of isopropyl alcohol (IPA) in the ball milling process resulted in lubrication, which minimizes the fierceness of the shocks. On one hand, graphite particles were peeled off to platelets. On the other hand, Si nanoparticle agglomerates were prevented and these nanoparticles were placed in cavities and surrounding graphite. In previous works, ethanol was used to yield a more homogeneous deposition of silicon nanoparticles on the graphene sheets [29]. A backscattered image (Figure 3d) was also recorded for the s-BMW sample, supporting the homogeneous distribution of the silicon nanoparticles, which appear in form of glittering points along the entire electrode. Transmission electron microscopy (TEM) micrographs registered in dark field reveal the microstructure differences between s-BMD (Figure 3e) and s-BMW (Figure 3f) samples. While the former one is poorly crystallized and formed by agglomerates of small particles where the pristine lamellar structure of graphite is completely vanished, in the s-BMW sample crystalline, Si nanoparticles and graphite particles are still distinguishable, which has to be ascribed to the lower friction generated upon the wet route because of the addition of IPA.

**Figure 4:**
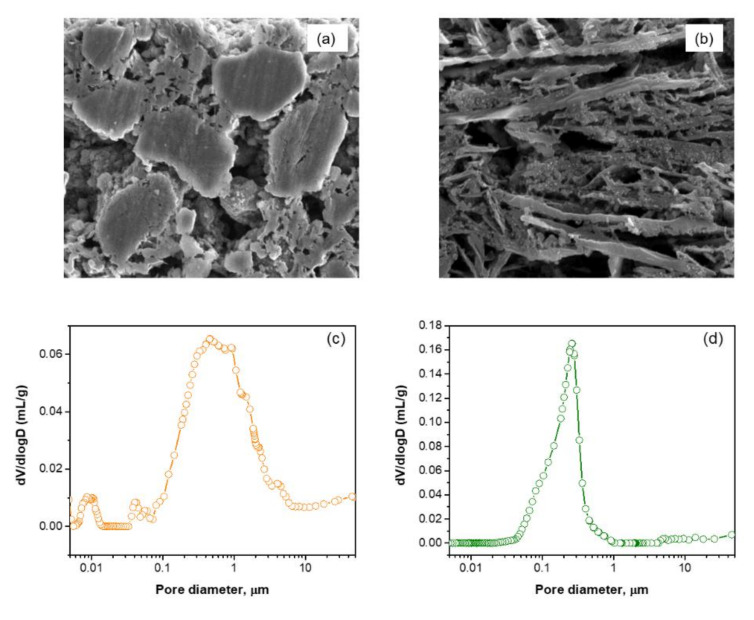
Cross section SEM images of pristine electrodes (**a**) e-BMD and (**b**) e-BMW. Mercury intrusion results of the pristine electrodes (**c**) e-BMD and (**d**) e-BMW.

A detailed structure of the pristine electrodes is shown in the SEM images (Figure 4). The electrode prepared using s-BMD as active material (hereafter denoted as e-BMD) reveals a microstructure formed by huge voids and spaces. In addition, aggregates of graphite platelets and Si as well as a globular morphology are detected (Figure 4a). However, the electrode prepared using s-BMW as active material (hereafter denoted as e-BMW) shows a very ordered electrode packing in which FLG flakes oriented parallel to each other predominate and where the Si nanoparticles are appropriately distributed in the electrode (Figure 4b). In order to better understand the distribution of Si nanoparticles in the electrode, the energy dispersive X-ray (EDX) mapping of pristine electrodes can be found in Figure S4. The SEM images of the pristine electrodes are in good agreement with the mercury intrusion results. The pore size distribution in e-BMD (Figure 4c) presents different regions in which there are pores with a diameter that varies from 6 to 0.5 μm, from 0.3 to 0.07 μm, and from 0.015 to 0.008 μm. As a consequence of this wide range of pore size distribution, more surface will be exposed to the electrolyte during the electrochemical reaction, which drives a continuous formation of SEI and causes continuous lithium losses. The mercury intrusion result for the e-BMW electrode (Figure 4d) shows a narrow range of the pore size distribution with a pore diameter ranging from 1 to 0.25 μm, which corroborates a better and more homogeneous electrode packing.

The main observation resulting from the physicochemical characterization of the Si@G samples is that an efficient dispersion and distribution of the particles are reached with the addition of IPA during the synthesis procedure. This process gives, as a result, an electrode that possesses optimal microstructure that gives to it the ability to mitigate the Si volume changes during cycling.

Additionally, since the two Si@G samples were not synthesized in the same conditions, a new Si@G sample was synthesized by following the same conditions and the same parameters as those used for the s-BMW with no addition of IPA. The SEM image (Figure S5a) shows that the morphology is very similar to that presented by s-BMD. Moreover, from the SEM cross-section image (Figure S5b), it can be appreciated how some voids and randomly orientated particles appear along the electrode, while a parallel particle’s orientation is maintained for the BMW electrode.

### 2.2. Electrochemical Behaviour

**Figure 5:**
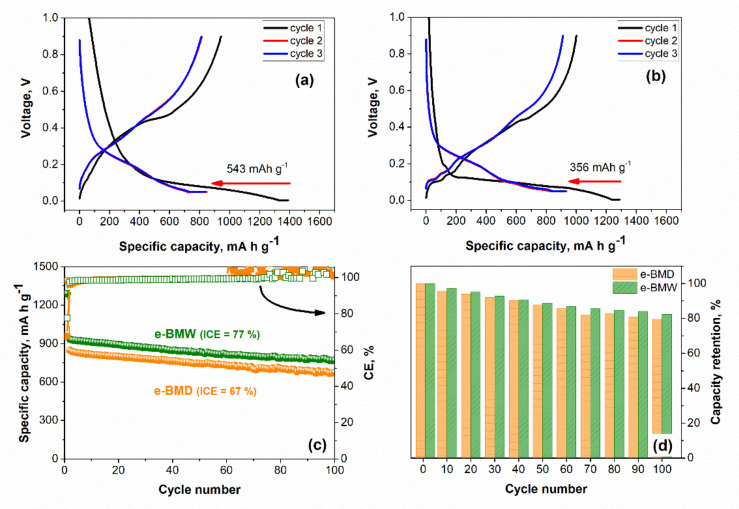
Discharge—charge curves from cycle 1 to 3 of (**a**) e-BMD and (**b**) e-BMW. The irreversible capacity of the first cycle is shown. (**c**) Coulombic efficiency (CE) (squares) and specific capacity (spheres) vs. cycle number of e-BMD (orange) and e-BMW (green). The initial coulombic efficiency (ICE) is shown. (**d**) Capacity retention of e-BMD (orange) and e-BMW (green). The voltage window is 0.05–0.9 V and the current density is 250 mA/g. First activation cycle: 0.005—0.9 V at 100 mA/g.

In this paper, the terms discharge and charge are referred to lithiation and delithiation processes, respectively. The specific capacities and current rates are given per mass of the active material (30% Si + 50% graphite). In all the electrochemical tests, a constant current/constant voltage (CCCV) step was applied during each lithiation cycle. Figure 5a shows the discharge-charge curves from the first to the third cycle of e-BMD cycled at 250 mA/g in the voltage window of 0.05–0.9 V. In order to achieve a better lithiation, the first cycle (formation cycle) was performed at 100 mA/g between 0.005 and 0.9 V. However, it has to be pointed out that, below 0.05 V, the metastable crystalline Li_15_Si_4_ (Li_3.75_Si) phase is formed. The de-lithiation reaction of this phase leads to the formation of amorphous Li_~2_Si, which causes particle cracking due to internal stresses and capacity fading [10,11]. For this reason, the voltage of subsequent cycles was fixed at 0.05 V. The specific capacity measured in the first discharge and charge is 1390 mA h/g and 943 mA h/g, respectively. In the second cycle, the discharge capacity decreases down to 847 mA h/g and, in the 100^th^ cycle, is ca. 674 mA h/g (Figure 5c). Since the final content of Si and graphite in the electrode is 30% and 50%, respectively, and, in order to compare the capacity values presented by the cells, the theoretical capacity of the Si@G anode was calculated by only considering the 30% of the theoretical capacity of Si and 50% of the theoretical capacity of graphite, giving rise to a value of 1259.7 mA h/g. The e-BMD first discharge capacity overcomes the theoretical one. This fact is related not only to the contribution of the SEI but also to the poorly crystallized character of e-BMD in agreement with the XRD, SEM, TEM, Raman, and mercury intrusion results. On the other hand, Figure 5b shows the discharge-charge curves from the first to the third cycle of e-BMW cycled in the same conditions than e-BMD. In this case, the plateaus are clearer than in e-BMD and various processes can be differentiated. In the first discharge, the formation of lithium graphite intercalation compounds takes place below 0.2 V [47]. In addition, this first discharge is dominated by the conversion process of Si to Li_~3.5_Si at 0.1 V. As described from other researchers [48], it is possible to distinguish three different processes during the second and third discharge: i) from 0.3 to 0.18 V, there is a gradual lithiation of Si lattice, ii) the formation of small Si clusters due to the breakup of the Si–Si bonds occurs from 0.18 to 0.09 V and results in the formation of isolated Si anions, and iii) the process corresponding to the region from 0.09 to 0.05 V is assigned to the lithiation of the isolated Si anions. During charge, the delithiation of graphite can be recognized below 0.3 V while the delithiation of Si is observed at potentials between 0.45 and 0.5 V. For a more detailed view of these lithiation/delithiation processes, the differential capacity plot of e-BMW is shown in Figure S6.

Regarding capacity values, the first cycle of e-BMW gives a specific capacity in the first discharge and charge of 1290 mA h/g and 1001 mA h/g, respectively. Then, capacity fades until 934 mA h/g and it is maintained close to 850 mA h/g for 100 cycles, as can be seen in Figure 5c. Yoon et al. [49] used the ball milling to reduce the size of the Si particles and to disperse the Si nanoparticles using ethanol. Then, they coated these nanoparticles into natural graphite. Their Si-coated graphite composite presented a first discharge capacity value of 761 mA h/g and the 78% of the capacity was retained at the 300th cycle. On the other hand, Maddipatla et al. [50] presented a Si/C anode material, which was prepared following a high energy milling step to produce nanoscale Si particles, a carbonization step, and a final high energy milling step of the Si/C-coated powders. The composite delivered a remarkable capacity of 1181 mA h/g at the 100^th^ cycle. Xu et al. [51] followed various ball milling steps with a final heating step to produce Si/graphite, Si/graphite/Cu, and Si/graphite/Cu/CNTs composites. The latter presented a reversible capacity of 646.5 mA h/g after 100 cycles at 0.2 A/g.

It has to be prompted that, during the synthesis step, a slight amount of SiO_2_ was formed on the silicon surface, as shown from XRD and XPS results. When SiO_2_ reacts with lithium, electrochemically inactive phases such as Li_2_O and Li_4_SiO_4_ are irreversibly formed. In some cases, they can buffer the volume changes experimented by silicon during cycling, which enhances the cycling performance [52]. However, the presence of these irreversible phases can also lead to a decreasing of the capacity values since less Si is available. Taking into account that the SiO_2_ content is higher when the dry route is followed, it would explain that e-BMD presented lower capacity values than e-BMW. In addition, some studies reported better capacity values when the thickness of the SiO_2_ layer in the Si anodes was reduced [53,54].

Additionally, the initial coulombic efficiency (ICE) is improved in e-BMW with a value of 77% when compared to 67% presented in e-BMD (Figure 5c). The lower ICE in e-BMD is attributed to its microstructure, as it was previously described in this work, due to the wide range of pore size distribution in e-BMD. More surface is exposed to the electrolyte during the electrochemical reaction. This fact can lead to a continuous formation of SEI. Consequently, ICE value is lower in e-BMD. Notably, CE values during cycling are more stable in e-BMW (>99%). Graphite milling leads to a better intercalation kinetics as well as a better electrolyte penetration into the material providing high stability and capacity retention [55]. However, in e-BMD, the pulverization of graphite as consequence of the high energy applied to the material during the milling process seems to lead to the graphitic structure collapse during cycling, which is not able to withstand the expansion of the Si nanoparticles. From cycle 1 to 60, it seems that the voids already presented by the microstructure are able to accommodate this expansion but, when cycle 60 is reached, the anode structure collapses and some compounds that form the SEI can be released. They migrate to the metallic lithium by reacting and giving rise to parasitic reactions, which make the capacity value during charge higher than that during discharge. Thus, it would explain why the CE is above 100% from cycle 60 until cycle 100. Figure 5d shows the capacity retention of e-BMW and e-BMD. While e-BMW was able to run 100 cycles and retain above 80% of the capacity, e-BMD presented values below this percentage in the 100th cycle.

**Figure 6:**
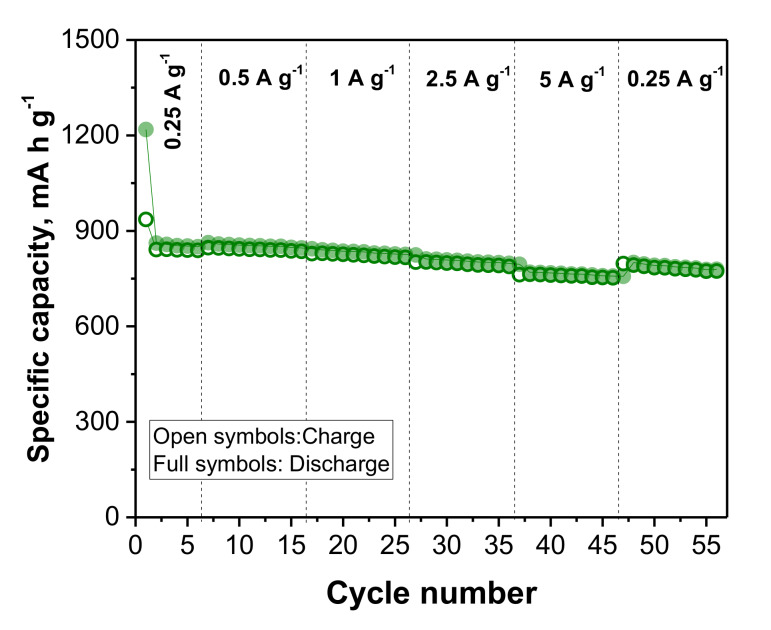
Rate performance of e-BMW. Voltage window: 0.05–0.9 V. 1^st^ activation cycle: 0.005–0.9 V at 100 mA/g.

Until this point, differences between samples in terms of capacity values, coulombic efficiencies, and capacity retention have been detected. One of the requirements for a full cell system is the selection of an anode, which is able to reach at least 100 cycles with good stability and capacity retention as well as stable coulombic efficiencies. Thus, e-BMD was discarded at this point of the work due to its poor electrochemical properties. Lastly, to complete the study, the rate performance of e-BMW was evaluated at 0.25, 0.5, 1, 2.5, and 5 A/g, as can be seen in Figure 6. Remarkably, e-BMW exhibits an excellent capacity retention with capacity values of 862, 860, 850, 820, and 770 mA h/g when cycling at 0.25, 0.5, 1, 2.5, and 5 A/g, respectively. Meanwhile, returning from 5 and 0.25 A/g, the BMW anode shows a capacity value closed to 800 mA h/g, which demonstrates very good reversibility. Tie et al. [56] synthesized a Si@SiO@GNS (graphene nanosheets) composite through ball milling of Si nanoparticles and expanded graphite at 500 rpm for 15 h. Their composite showed capacity values of 1400, 700, and 400 mA h/g at 0.2, 1, and 2 A/g, respectively. On the other hand, in a recent work of Zhao et al. [57], porous silicon@carbon composites were obtained through ball milling at 200 rpm for 2 h, among other polymerization and sulfur-melting processes. The anode showed a capacity of 1178 mA h/g at 0.2 A/g and a capacity of 751 mA h/g at 1 A/g. Compared to other research studies [49,50,51,57], the Si@graphite material used in this case is synthesized in a single step that is easily-scalable, without further steps for reducing the size of the particles, since we used Si nanoparticles, nor for heating powder treatments.

**Figure 7:**
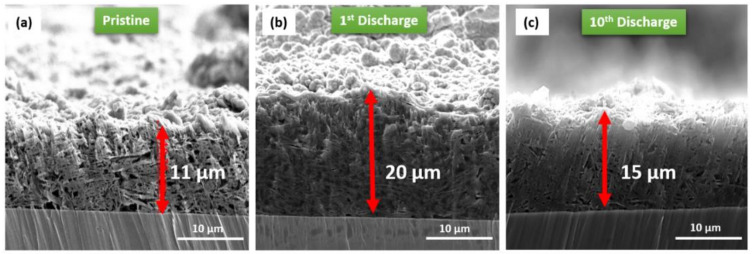
Cross section SEM images of the e-BMW ion milled electrodes (**a**) pristine (**b**) after the 1st discharge (0.005 V) and (**c**) after the 10^th^ discharge (0.05 V).

The reasons of the satisfactory performance of the rate capability test are: (i) suitable distribution of particles and intimate contact between them, (ii) the presence of small Si nanoparticles (<50 nm) in the anode implies shorter diffusion paths for Li ions, which means the current can be increased, and (iii) the use of FLG as a conductive additive in the anode formulation seems to play an important role reinforcing the anode microstructure due to its ability to buffer Si volume changes. To strengthen these points, SEM cross section images after lithiation were recorded. In pristine e-BMW (Figure 7a), very good mixing between components is reached and agglomeration is prevented, which leads to a more intimate contact between particles. In addition, the FLG flakes are observed as being oriented parallel to each other. The thickness of the electrode is 11 μm. After the first lithiation (voltage = 0.005 V), the SEM cross section image (Figure 7b) shows that the thickness is twice the pristine electrode. The FLG flakes are preserved even though they are less visible due to the volume growth of Si that causes particle stacking. Lastly, after the tenth discharge (voltage = 0.05 V) (Figure 7c), there are no significant changes in thickness being slightly higher than the pristine electrode. In addition, no delamination is detected and FLG flakes are still present. The difference in thicknesses between the electrodes during the first and tenth discharge can be explained as follows. The first discharge was performed at a cut-off voltage of 0.005 V and corresponds to the activation cycle, which leads to the formation of an over-lithiated phase (Li_3.75_Si). The volume expansion experimented by silicon nanoparticles during the first lithiation process leads to an increase of the electrode thickness. However, the following discharges are performed at a higher cut-off voltage (0.05 V), which drives a less lithiated phase. Therefore, the volume expansion is bigger when the over-lithiated phase Li_3.75_Si is formed. Thus, the electrode thickness in the first discharge is thicker than that in tenth discharge.

Therefore, achieving an optimal microstructure at the electrode level is crucial for preserving the morphology and preventing not only fractures but also delamination from the current collector.

## 3. Materials and Methods

### 3.1. Material Preparation

For the preparation of Si@G samples, the ball milling route was followed. Silicon nanoparticles (Alfa Aesar, Ward Hill, MA, USA) were mixed with graphite (SFG15L, Imerys, Paris, France) in a weight ratio of 37.5: 62.5 in a planetary mill (Pulverisette, Fritsch, Idar-Oberstein, Germany) at 1000 rpm for 10 min. For comparison, the wet route was also followed by adding 10 mL of IPA (Scharlab, Barcelona, Spain). In this case, the boiling point of the solvent (82.5°) can increase the pressure generated inside the bowl. Consequently, the speed was set up to 400 rpm. The milling time was 2 h with pauses for cooling. In both cases, zirconia bowls (the 50% of the space in the bowls was left empty) and YSZ (Yttria stabilized zirconia, Inframat, Manchester, CT, USA) balls with a diameter of 0.5 mm were used. Considering the addition of IPA, in order to make comparable both methods (dry and wet), the number of balls was adjusted. This resulted in 180 balls employed in the dry route and 250 balls employed in the wet one.

### 3.2. Electrode Preparation

The electrodes were processed according to the formulation 80 wt.% of active material, 10 wt.% of conductive additive, and 10 wt.% of binder. The percentage of Si in the active material is 30. Powdered FLG (UCAM) and lab made LiPAA were used as conductive additive and binder, respectively. The materials mentioned were mixed in an IKA’s Ultra-Turrax (IKA, Staufen, Germany) at 6000 rpm for 1 h using distilled water as a solvent. Lastly, the obtained slurry was uniformly coated on copper foil using a doctor blade. Once dried, the electrodes were punched with a diameter of 12 mm and vacuum dried at 120 °C overnight. The final loading of all the electrodes was 1 mg cm^−2^.

### 3.3. Characterization

For the sample characterization, XRD patterns were recorded in a Bruker D8 Discover diffractometer (Billerica, MA, USA) using Cu-Kα radiation source. SEM measurements were performed in a FEG Quanta 200 from ThermoFischer (Waltham, MA, USA). TEM was carried out in a FEG Tecnai G2 F20 from ThermoFischer, operated at 200 keV. XPS was measured in a Phoibos 150 XPS spectrometer (SpecsGroup, Berlin, Germany) with non-monochromatic Mg Kα radiation (1253.6 eV). The scans were collected at high-resolution (energy step = 0.1 eV, energy pass = 30 eV) at low power (100 W). The binding energies of the spectra were calibrated to the C1s peak at 284.8 eV. Raman spectra were recorded with a Renishaw spectrometer (Nanonics Multiview 2000, Jerusalem, Israel) operating with an excitation wavelength of 532 nm. Mercury intrusion experiments were executed using an AutoPore V (Micromeritics, Norcross, GA, USA). The range of pressure applied varied from 3 to 61000 PSI. For the ex-situ SEM measurements, the electrodes were ion beam milled at 80 °C and an angle of 90° (Ion Milling 4000Plus, Hitachi, Tokyo, Japan) in order to obtain an undistorted cross-section milling.

### 3.4. Electrochemical Testing

The electrochemical measurements were performed in CR2032-type coin cells assembled inside a glovebox under an argon atmosphere. The half cells were assembled using Si@G as a positive electrode, a disc of metallic lithium as the negative one, and a Whatman glass fiber disc as a separator of both electrodes. 1M of lithium hexafluorophosphate (LiPF6) in FEC + ethyl methyl carbonate (EMC) (FEC: EMC = 3:7 wt. %) + 2% VC (Soulbrain MI, Northville, MI, USA) was used as electrolyte solution. Galvanostatic experiments were run in a MACCOR battery tester between 0.05–0.9 V at different current rates. The stability of the cells was evaluated from the discharge/charge curves obtained using a CC/CV method at 0.020 mA/0.005 V for the first cycle and at 0.020 mA/0.05 V for the following cycles.

## 4. Conclusions

In this work, we have followed a simple, low-cost, and easily scalable approach for fabricating Si@G electrodes with a 30% of Si in their formulation to be used as anodes in lithium ion batteries. The characterization and electrochemical results altogether suggest that the wet ball milling conditions used here are optimal for fabricating an anode with not only capacity values up to 850 mA h/g but also an excellent stability and capacity retention even at high current density (5 A/g). From the results, we found the strong impact that the synthesis procedure has on the anode microstructure and morphology. An efficient dispersion and distribution of the particles are reached with the addition of IPA during the synthesis procedure and, together with the use of FLG as a conductive agent, result in an electrode with an optimal microstructure that is able to mitigate Si volume changes during cycling. To summarize, the Si@G anode synthesized in this work through the wet ball milling process is positioned as a promising candidate for mitigating the problem of high-power and high-energy applications. These results encourage us to investigate its application in a full cell device.

## Supplementary Materials

The following are available online. Table S1: Data results of the Si diffraction peak at 28°, Figure S1: XRD pattern of the dry milled graphite (M-G), Figure S2: Raman spectrum of the dry milled graphite (M-G), Figure S3: SEM images of graphite and dry milled graphite (M-G), Figure S4: EDX elemental mapping of pristine electrodes, Figure S5: SEM image of Si@G synthesized following the wet conditions without the addition of IPA and SEM cross section image of the corresponding electrode, Figure S6: Differential capacity profile of e-BMW.
