# Supplementary material for: Towards a High-Power Si@graphite Anode for Lithium Ion Batteries through a Wet Ball Milling Process
Source: Molecules. 2020 May 27;25(11):2494. doi: 10.3390/molecules25112494 (PMC7321121; doi:10.3390/molecules25112494)
Supplement: Supplementary file 1 [file molecules-25-02494-s001.pdf]

Towards a high-power Si@graphite anode for lithium ion batteries through wet ball milling process

Marta Cabello*[1], Emanuele Gucciardi[1], Alvaro Herrán[1], Daniel Carriazo[1,2], Aitor Villaverde[1], Teófilo Rojo[1,3]

[1] Centre for Cooperative Research on Alternative Energies (CIC energiGUNE), Basque Research and Technology Alliance (BRTA) Parque Tecnológico de Álava, Albert Einstein 48, 01510 Miñano, Álava, Spain

[2] IKERBASQUE, Basque Foundation for Science, 48013 Bilbao, Spain

[3] Inorganic Chemistry Department Faculty of Science and Technology University of the Basque Country UPV/EHU, 48080 Bilbao, Spain

*: E-mail: mcabello@cicenergigune.com
Telephone: (+34) 945297108

	Crystallite size (nm)	Lattice strain (%)
Si	37.05	-
BMD	14.09	0.143
BMW	35.45	0.011

Table S1: Data results of the Si diffraction peak at 28°. The crystallite size has been obtained using the Scherrer formula considering the FWHM.

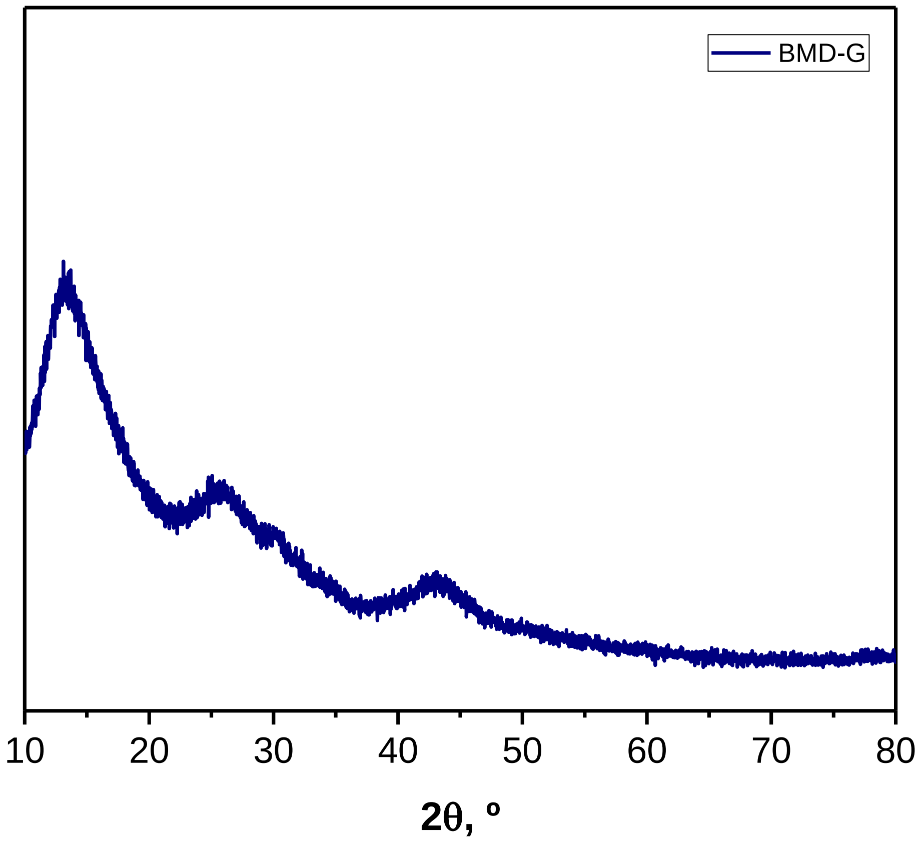

Figure S1: XRD pattern of the dry milled graphite (M-G). The broad band between 10-20º corresponds to the sample holder.

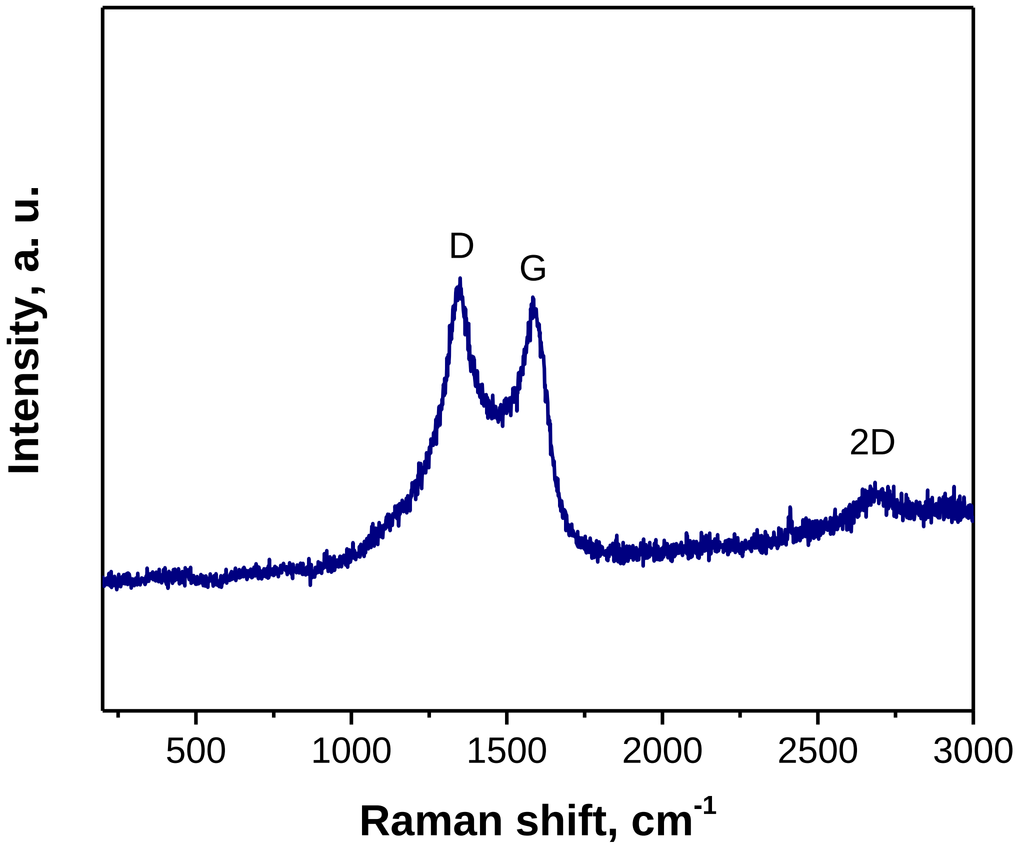

Figure S2: Raman spectrum of the dry milled graphite (M-G)

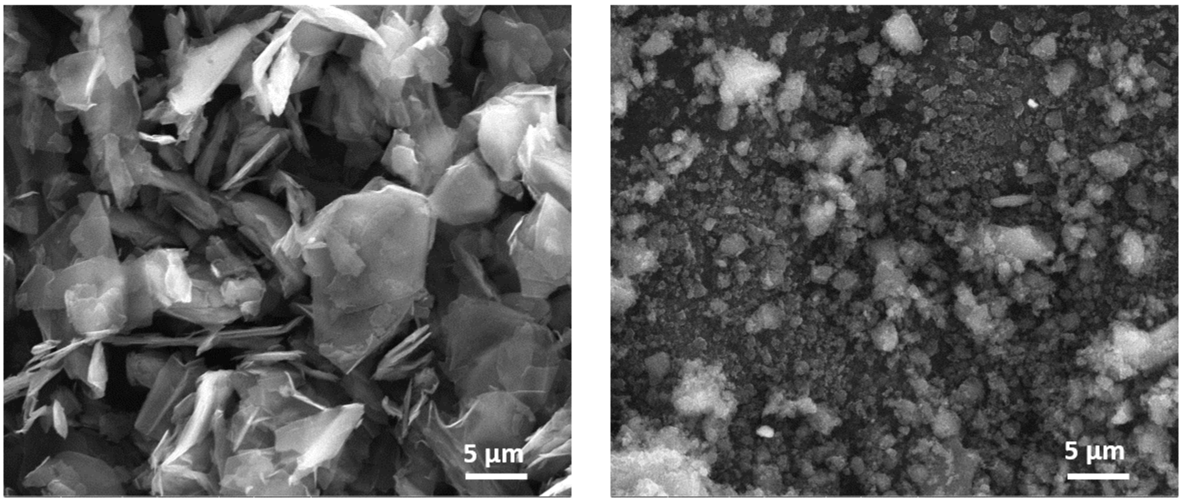

Figure S3: SEM images of graphite (left) and dry milled graphite (M-G) (right)

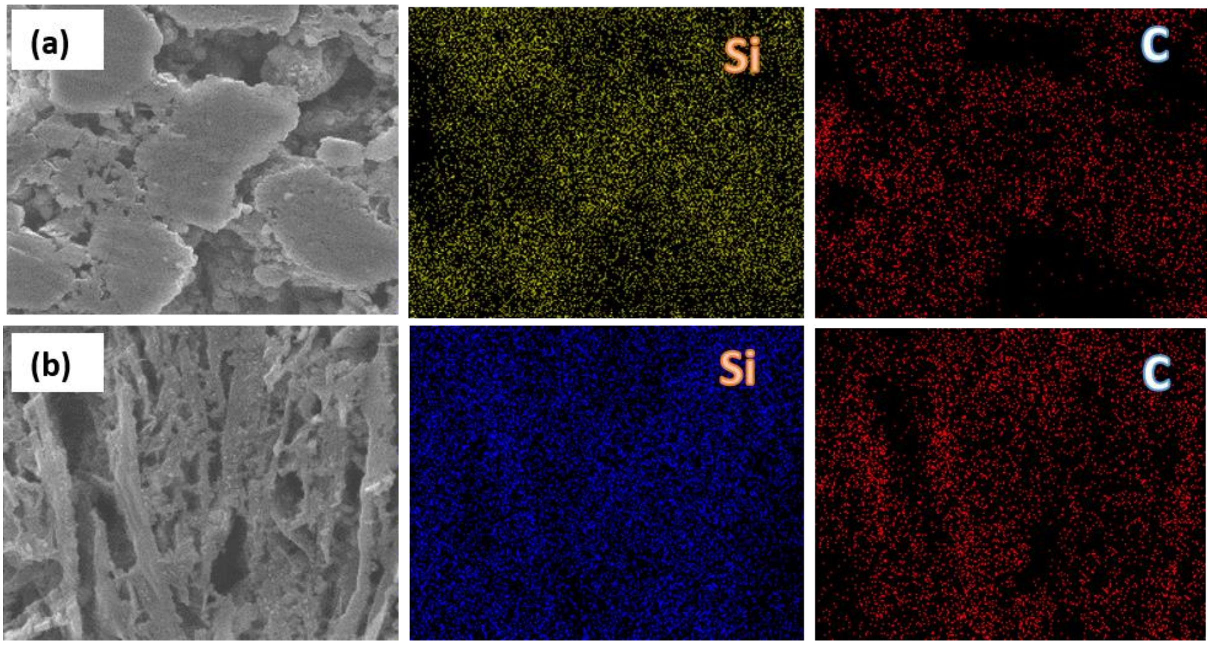

Figure S4: EDX elemental mapping of pristine electrodes of a) e-BMD and b) e-BMW.

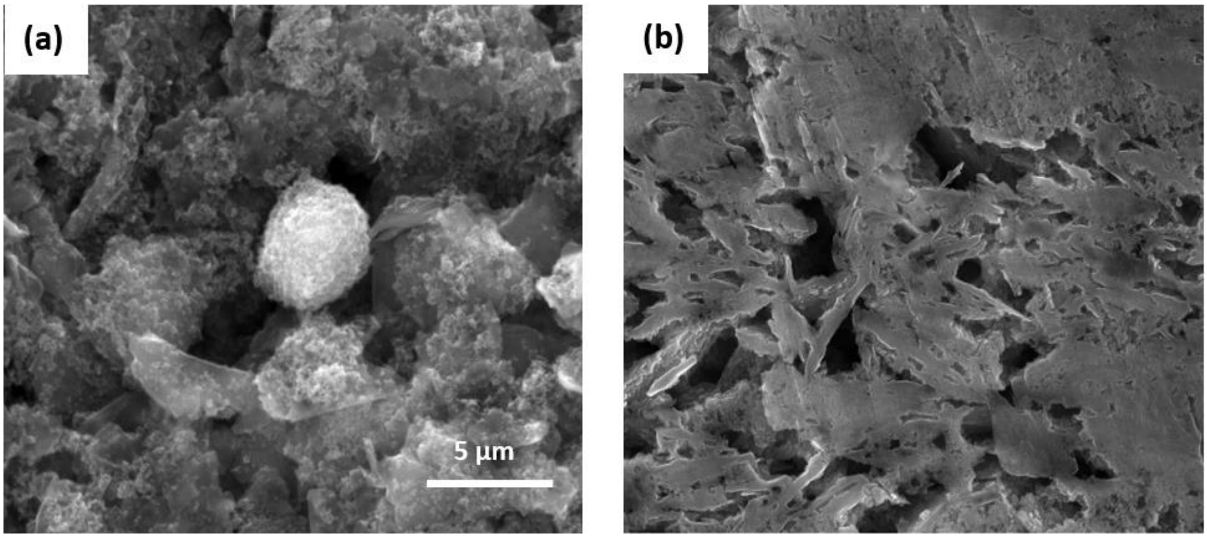

Figure S5: a) SEM image of Si@G synthesized following the wet conditions without the addition of IPA. B) SEM cross section image of the corresponding electrode. The thickness of the electrode is 28 µm.

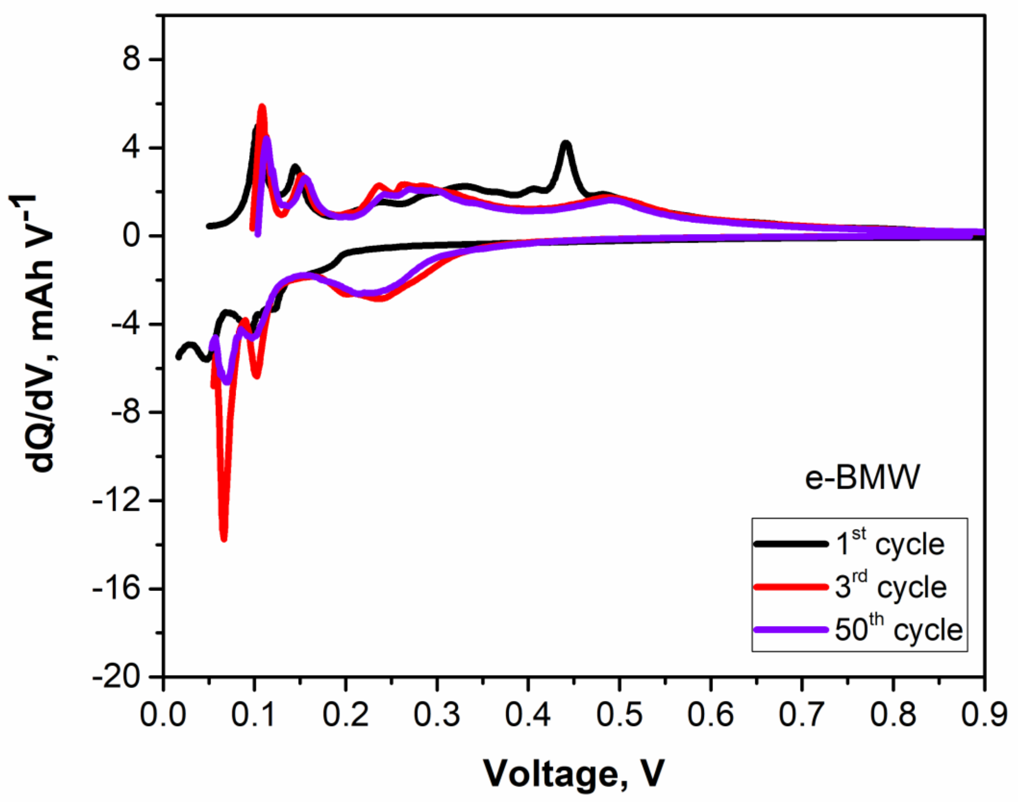

Figure S6: Differential capacity profile of e-BMW. The voltage window is 0.05 – 0.9 V and the current density is 250 mA g^{-1}. 1st activation cycle: 0.005 – 0.9 V at 100 mA g^{-1}.
